# Light Emission Intensities of Luminescent Y_2_O_3_:Eu and Gd_2_O_3_:Eu Particles of Various Sizes

**DOI:** 10.3390/nano7020026

**Published:** 2017-01-25

**Authors:** Jens Adam, Wilhelm Metzger, Marcus Koch, Peter Rogin, Toon Coenen, Jennifer S. Atchison, Peter König

**Affiliations:** 1 INM—Leibniz Institute for New Materials, 66123 Saarbrücken, Germany; jens.adam@leibniz-inm.de (J.A.); marcus.koch@leibniz-inm.de (M.K.); peter.rogin@leibniz-inm.de (P.R.); jennifer.atchison@leibniz-inm.de (J.S.A.); 2 Siemens AG, Corporate Technology, 81739 Munich, Germany; wilhelm.metzger@siemens.com; 3 DELMIC B.V., 2629JA Delft, the Netherlands; coenen@delmic.com

**Keywords:** Y_2_O_3_:Eu, Gd_2_O_3_:Eu, luminescence, fluorescence, cathodoluminescence, scintillation, particle size effect

## Abstract

There is great technological interest in elucidating the effect of particle size on the luminescence efficiency of doped rare earth oxides. This study demonstrates unambiguously that there is a size effect and that it is not dependent on the calcination temperature. The Y_2_O_3_:Eu and Gd_2_O_3_:Eu particles used in this study were synthesized using wet chemistry to produce particles ranging in size between 7 nm and 326 nm and a commercially available phosphor. These particles were characterized using three excitation methods: UV light at 250 nm wavelength, electron beam at 10 kV, and X-rays generated at 100 kV. Regardless of the excitation source, it was found that with increasing particle diameter there is an increase in emitted light. Furthermore, dense particles emit more light than porous particles. These results can be explained by considering the larger surface area to volume ratio of the smallest particles and increased internal surface area of the pores found in the large particles. For the small particles, the additional surface area hosts adsorbates that lead to non-radiative recombination, and in the porous particles, the pore walls can quench fluorescence. This trend is valid across calcination temperatures and is evident when comparing particles from the same calcination temperature.

## 1. Introduction

Yttrium oxide doped with europium (Y_2_O_3_:Eu) is a luminescent material with red-orange emission upon UV excitation (fluorescence), excitation by electrons (cathodoluminescence), and excitation by X-rays (scintillation). The versatility of excitation modes has made this material a very compelling choice for display technologies and fluorescent lamps. Depending on the application the phosphor particles can be micron sized (lamps, cathode ray tube (CRT) screens) [1] or down to nanometre sized for high resolution plasma displays [2]. Therefore, a study that characterizes a range of particle sizes synthesized using the same wet chemistry techniques and with the same calcination temperatures to determine the effect of the particle size on luminescence efficiency using all three excitation sources is technologically interesting.

In addition, yttrium can be replaced partially or fully by gadolinium without changing the cubic RE_2_O_3_ crystal structure in a continuous solid solution system and without changing the luminescence properties, conferring the advantage of improved X-ray absorption because of the larger atomic mass of Gd. When comparing the relative light output of (Gd,Y)_2_O_3_:Eu to other sintered ceramic scintillators, it is the third best material after CsI:Tl and doped Gd_2_O_2_S [1,3] and it is the best material with a simple oxide character. Its synthesis is straight forward and the average particle size can easily be controlled, making it a good model system for studying size effects.

In the display technology field, smaller particles are expected to improve resolution by combining the field emission of electrons with the cathodoluminescence of the particles [4,5,6,7,8,9,10]. For particles less than 10 nm in diameter, the possibility of exploiting quantum confinement effects with their respective changes in electron transitions and spectral details [2,11,12] have been reported. Moreover, colloidal processing allows the production of deagglomerated nanoparticles to obtain fluorescent transparent dispersions that have applications such as security marks, as well as biomarkers [4,13,14,15,16,17]. Some of these cited studies hint that small particles emit more light than large ones, however, these studies are usually based on the comparison of only two [7] or very few particle batches [18] and single excitation methods. In contrast, more fundamental studies report that smaller particles have lower emission intensity [3,4,9,19,20]. Specifically, Jing et al. have shown a clear trend in Eu doped yttrium oxide particles in a size range from 100 to 900 nm and have compared them to 3 µm commercial particles under electron beam excitation to show increasing luminescence with increasing particle size [21]. However, besides inner particle effects, overall optical mechanisms such as scattering in particle packing have to be considered before assessing the performance of particulate phosphor screens [3,4,21].

The phosphor coatings of lamps, CRTs, and of cathodoluminescence or X-ray intensifying screens are usually made of micron-sized particles [1,3]. Particles of this size are commonly made by simple solid state reactions during calcination. Alternatively, nanometre and sub-µm sized particles are accessible by wet chemical synthesis. To synthesize small (Y,Gd,Eu)_2_O_3_ particles, precipitation chemistries can be found in the literature. Specifically, there is the urea based homogeneous precipitation (UBHP) that uses a continuous shift, of a full or partial aqueous reaction medium, to the alkaline range leading to very well defined seed formation and particle growth [9,10,13,17,22,23,24]. Another common method is the merging of mainly aqueous RE-salt solutions with an alkaline solution leading to an instantaneous precipitation [8,16,18,20,25]. Two other common synthesis approaches are the polyol-mediated [12,26,27,28] and the solvothermal [5,6] wet chemical syntheses. In this study, we have modified the instantaneous precipitation reaction using Na_2_O_2_ as a new compound for the alkaline solution (peroxidic reaction) and use the UBHP reaction as described in the literature.

An advantage of synthesizing small particles is that they can be prepared by colloidal processing which can be a precursor for wet coating processes that yield well defined thin coatings. In addition, complex lateral structures can be made by printing inks or pastes, which are exploitable in printed electronics. For example, Büchele et al. followed such a hybrid approach to enhance X-ray imaging using an organic photovoltaic matrix embedded with Gd_2_O_2_S:Tb particles for the scintillation functionality [29].

These syntheses and processing routes produce different sized particles, however, the effective use of these particles in functional composites, such as in printed electronics, requires a better understanding of how their physical properties change with particle size.

It is the aim of this study to provide and discuss in-depth the experimental results on the dependence of the emitted light intensity on the size of luminescent particles made of (Y,Gd,Eu)_2_O_3_ using three different excitation modes. We aim to characterise particles of a broad size range. This was realised by variations of the synthesis conditions and by including optimised commercial lamp phosphor particles as a reference for the study. Structural particle properties are characterised in detail to reveal morphological influences on the results.

## 2. Experimental Procedure

### 2.1. Materials

- Y(NO_3_)_3_·6H_2_O, batch ‘YN1/13’, purity 99.9%, Treibacher Industrie AG, Althofen, Austria
- Gd(NO_3_)_3_·6H_2_O, batch ‘23989500’, purity 99.9%, Strem Chemicals, Kehl, Germany
- Eu(NO_3_)_3_·6H_2_O, batches ‘23118500’ and ‘21422100’, purity 99.9%, Strem Chemicals, Bischheim, France
- Na_2_O_2_, batch ‘MKBP0012V’, purity 97%, Sigma Aldrich Chemie GmbH, Steinheim, Germany
- Urea, CH_4_N_2_O, batch ‘SZBD2810V’, purity 99%, Sigma Aldrich Chemie GmbH, Steinheim, Germany
- Micron sized Y_2_O_3_:Eu (4 mol %), labeled ‘L581’, a commercial phosphor from Osram (a former Siemens subsidiary) for use in fluorescent lamps. It is used as a standard because it has optimized emission intensity in the orange-red range when excited by a mercury vapour lamp at 254 nm with a reported quantum yield of 85%

### 2.2. Peroxidic Precipitation (PP)

The PP batches were prepared by mixing non-calcined powders from two or three identical precipitation steps. Each precipitation proceeded with precursor amounts resulting in a theoretical oxide yield after calcination of between 12 and 18 g and resulting in a doping level of 5 mol % Eu_2_O_3_ with respect to the entire oxide. The metal nitrates were dissolved at 0.22 mol/L for the Y_2_O_3_:Eu samples or at 0.10 mol/L for the Gd_2_O_3_:Eu and (Y_0.5_Gd_0.5_)_2_O_3_:Eu samples in deionised water. The Na_2_O_2_ was dissolved at 0.30 to 0.33 mol/L in deionised water. Then the nitrate solution was poured quickly into the Na-peroxide solution and stirred overnight. The following day the solution was heated to a temperature of 90 °C and held for one hour under continued stirring.

### 2.3. Urea Based Homogeneous Precipitation (UBHP)

Each precipitation was conducted with precursor amounts resulting in a theoretical oxide yield of typically 14 g and resulting in a doping level of 6 mol % Eu_2_O_3_ with respect to the entire oxide. Although several studies suggest that around 2 wt % is the optimum doping level for cathodoluminescence, for this study we chose 6 wt % to accommodate all the excitation sources [2]. Only Gd_2_O_3_:Eu was prepared following the UBHP precipitation method for this study. The urea and metal nitrates were dissolved in deionised water or in mixtures of deionised water and other solvents. The metal nitrate concentrations were varied between 0.010 and 0.015 mol/L. Table 1 lists the ratios of solvents and the urea concentrations used to control the size of the particles.

After dissolving nitrates and urea, the solution was stirred continuously for two hours. Then, the solution was heated to 85 °C and stirred at this temperature for 90 min except for NS133: in this case the solution was maintained at 85 °C for 240 min after the precipitate appeared.

### 2.4. Treatment of the PP and UBHP Precipitates

After each precipitation step, the aqueous reaction medium with the stirred precipitate was allowed to cool down to room temperature. The particles were washed by repeated centrifugation, decanted, and stirred to homogenise the precipitate sediment in fresh deionised water until a conductivity of <50 µS/cm was reached, and were then freeze dried. The PP particles were calcined in air for one hour at five different temperatures (450, 600, 700, 850, 1000 °C) in ceramic crucibles and all UBHP particles were calcined in air for six hours at 850 °C. After calcination, the powder samples were ground in a mortar to break up any large aggregates. All the calcined samples were subjected to the characterizations described below. The samples are named according to the scheme: NS[batch no.]-[calcination temperature/°C], e.g., NS135-700.

### 2.5. Physical Particle Properties—Morphology and Size Information

The specific surface area (SSA) of each particle sample was determined by N_2_ adsorption measurements and according to the BET (Brunauer, Emmett, and Teller) theory using an Autosorb AS6, Quantachrome (Boynton Beach, FL, USA). The analytical relationship between diameter (*d*_SSA_), density (ρ), and specific surface area (SSA) of monodispersed spheres with smooth surface is given by Equation (1):

*d*_SSA_ = 6/(SSA·ρ) (1)

This assumes the particles are ideal; in this study, the particles depart from ideality. The particles from both synthesis routes are spherical or equiaxed, however, the PP particles are aggregated and the UBHP particles have a rough surface and are porous. As a result, there are discrepancies between the *d*_SSA_ values and the particle diameters measured by electron microscopy. The *d*_SSA_ provides a simple parameter from which valuable size information can be extracted because it is an ensemble average over 100 mg of powder in contrast to environmental scanning electron microscope (ESEM) which is a local average of a few particles.

Environmental scanning electron microscope (ESEM). The Quanta 400 ESEM FEG, (FEI Europe, Eindhoven, the Netherlands) was operated at low vacuum mode at 100 Pa without conductive coating on the particles. A large field detector (LFD) was used to collect secondary electrons at 5, 10, and 20 kV accelerating voltage. The size *d*_SEM_ was obtained by averaging the size of at least 40 particles imaged by the ESEM.

Focused Ion Beam (FIB) Versa 3D Dual Beam (FEI Europe, Eindhoven, the Netherlands). Platinum was deposited onto the surface before FIB preparation. A trench was cut with a 30 kV Ga-ion beam at high beam currents and then the cross section was milled stepwise with lower beam currents under high vacuum conditions.

Transmission Electron Microscope (TEM). The JEM-2100, (JEOL, Akishima, Japan) was operated with LaB_6_ cathode at 200 kV. A drop of particle dispersion was placed on a holey carbon TEM grid (S147-4, Plano, Wetzlar, Germany) and dried in air before investigation. The particle size, *d*_TEM_, was obtained by averaging the size (measured with the help of a graphics program) of at least 30 particles imaged by the TEM.

X-ray diffraction (XRD). An Advance D8 diffractometer, Lynxeye detector, and Topas analysis software (Bruker, Karlsruhe, Germany), with a Cu-target, and the Kα spectral line was used to collect the X-ray reflections. The Topas software utilizes Rietveld refinement to determine the crystallite size, *d*_XRD_, according to the Scherrer correlation.

### 2.6. Luminescence Properties of the Particles

#### 2.6.1. Fluorescence and Quantum Yield (QY) under UV Excitation

Fluorescence spectra were taken with a Hitachi F-7000 fluorescence spectrophotometer (Hitachi, Tokio, Japan). For both the fluorescence spectra and the QY measurements the powders were placed in the flat quartz cuvette provided for QY measurements. For the fluorescence measurement, a flat cuvette was fixed into the solid sample holder. Photoluminescence (PL) spectra were taken with an excitation wavelength of 250 nm (Ex-Slit 20 nm, Em-Slit 1 nm resolution) and corrected for the instrumental response. Photoluminescence excitation (PLE) spectra were taken at an emission wavelength of 612 nm (Ex-Slit 1 nm, Em-Slit 20 nm resolution) without spectral correction.

For the characterisation of the absolute quantum yield, the original F-7000 Hitachi quantum yield measurement unit including the QY software for the calculation was used. This accessory consists of a 60 mm integrating sphere which is placed into the spectrophotometer with its centre at the crossing point of the excitation and detection light paths. For measurement, the flat QY quartz cuvette with the powder sample is placed in the direct (using a port opposite to the excitation light entrance) and indirect position (opposite to detector) on the outside of the integrating sphere. The measurement at the indirect position is used to correct the result concerning the excitation by initially non-absorbed and reflected excitation light which is redirected to the sample by the integrating sphere. Additionally, a measurement of Al_2_O_3_ powder is required to get the full amount of excitation light which is reflected by a non-absorbing sample. For the measurements, the excitation wavelength is set to 250 nm (Ex-Slit 5 nm, Em-Slit 5 nm resolution) and spectral corrections are used concerning the instrumental response and the integrating sphere. For the QY calculation, the excitation wavelengths 225–275 nm and the emission wavelengths 565–725 nm are considered.

In every QY session, we measured L581 and calculated its value. The average QY based on 7 measurements is (85.6 ± 1.0)%, which agrees with the manufacturer’s data. To determine the deviation for the samples with a smaller QY value, sample NS135-700 was measured 4 times and the standard deviation was calculated to be (14.9 ± 0.4)%.

#### 2.6.2. Light Yield under X-Ray Excitation (Scintillation)

**Figure 1:**
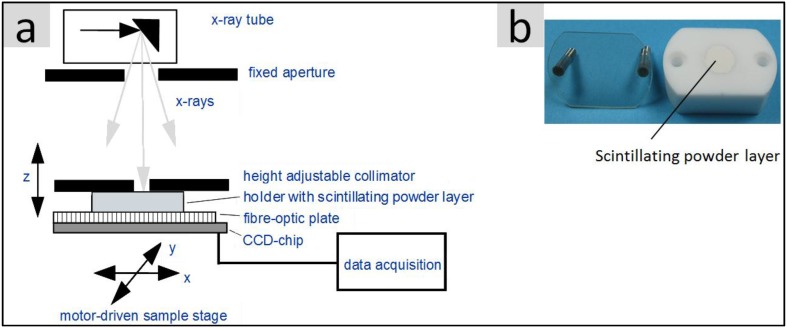
(**a**) Schematic structure of the X-ray measuring station for the spatially resolved determination of the X-ray excited light yield of scintillating materials; (**b**) Sample holder made of highly reflective Teflon containing a 0.5 mm thick scintillating powder layer (see the text for further details).

With regards to potential medical imaging applications, the light yield of the synthesized powders under X-ray excitation is an essential property. Figure 1a illustrates the test setup which was used for the characterization of the various scintillating materials. A special sample holder (Figure 1b) made of highly reflecting Teflon contains the respective powder, which is pressed into a shallow cylindrical cavity (10 mm diameter) and is covered by a glass plate which in turn can be fixed to the Teflon body by two screws.

The holder is placed top down directly onto the light detecting charge-coupled device (CCD)-chip which is equipped with a fibre-optic plate for radiation and mechanical protection reasons. The low thickness (0.5 mm) of the strongly scattering powder layer surrounded by reflecting Teflon walls ensures that a large part of the scintillating light reaches the photodetector. A commercial X-ray tube (Siemens SR 100/20) operated with a fixed emission spectrum (100 kV with 3 mm Al filter) and a fixed dose (4 mAs) is used as the excitation source. In order to get well defined excitation conditions, the sample was irradiated by a collimator (diameter 1.3 mm) which was located directly above the sample holder. The light distribution on the light exit side of the powder layer was subsequently integrated within a fixed region of interest, ROI (5 × 5 mm^2^). After correction for the spectral sensitivity of the Si-CCD-chip, the integrated intensity value corresponds to the light yield of the respective powder. A correction for different X-ray absorption was not implemented.

#### 2.6.3. Light Yield and Spectra Obtained under Electron Beam Irradiation (Cathodoluminescence/SEM)

Sample preparation: Small particle amounts were homogenised with an aqueous solution of poly(vinylalcohol) (PVA) and poly(ethylene glycol) (PEG) (4:1) for a total binder concentration of 6 wt %. About 100–150 mg of particles and binder were dried and then axially pressed at 10 kN to pellets with a diameter of 5 mm. They were coated with carbon and affixed to metallic stubs with carbon tape.

The measurements were taken with a cathodoluminescence (CL) detection system (developed at the FOM institute AMOLF, Amsterdam, and commercialized by DELMIC, Delft, the Netherlands) which is mounted on an FEI XL30 SFEG scanning electron microscope [30,31].

In this system, the CL is collected by an aluminium paraboloid mirror after which it is redirected out of the SEM. There the CL emission is focused onto a fibre which is coupled to a spectrometer (Acton SpectraPro 2300) with a back-illuminated liquid-nitrogen-cooled CCD array (Spec 10, 1340 × 100 pixels, Princeton instruments, Trenton, NJ, USA.

The focal point of the mirror is aligned with electron beam impact position by a piezoelectric micropositioning system and checked by using a 2D CCD array (Pixis 1024B, Princeton Instruments, Trenton, NJ, USA) [30]. This enables quantitative comparison between samples as the procedure can be performed in a systematic and reproducible manner.

All the measurements were performed at 10 kV acceleration voltage using 15 or 42 pA current depending on the brightness of the sample. For the radiation efficiency comparison, 10 measurements per sample were taken at random positions. Each of these measurements was spatially averaged over an area of 22.5 × 30 µm^2^ by leaving the camera collection running while scanning the electron beam over that area. Typically the overall camera integration time was 10 ms but times up to 2 s were used because of the large variation in sample brightness. The spectrometer slit width was 600 µm in order to efficiently collect the light from the fiber and a grating with 150 lines/mm blazed for 500 nm was used to cover the whole spectral range from 400 to 900 nm.

Each of the spectra was normalised to compensate for different e-beam currents and different integration times of the camera. Then they were corrected for the instrumental response and the 10 spectra of each sample were averaged to one spectrum. For the brightest sample (L581) it was found that the main peak was cut due to detector saturation in the range between 609 and 617 nm. Only the data points affected by saturation were replaced by a peak shape calculated by multiplying the data points of the sample NS143-1000 (PP/Gd_2_O_3_:Eu) by 3.5. This method leads to a good overlap of the modelled peak and original peak for the non-saturated peak sides, thus it should be appropriate to reconstruct the peak maximum. Afterwards, the intensities were summarised for the data points in the range of 565 to 725 nm (as for the fluorescence QY calculation). The summation values were normalised to assign the value of L581 to 100%.

To obtain two example spectra with better spectral resolution the slit width was set to 100 µm and a grating with 1200 lines/mm was used, covering the spectral bandwidth between 577 and 636 nm. Again, 10 spectra for each sample were taken at 42 pA current and 1 or 2 s integration time. They were averaged to the two spectra as shown in the results section. In this case the instrumental response correction was not necessary as this is insignificant due to the small wavelength range.

## 3. Results and Discussion

### 3.1. Yield, Composition, and Specific Surface Area of the Precipitates

The practical yield of the dried precipitates was always less than the ideal yield predicted by the reaction equation. In addition, there were losses attributed to the washing steps (e.g., incomplete sedimentation), freeze drying, and general powder handling, as well as, mass lost during calcination at 1000 °C. It is also possible that there was incomplete reaction conversion. The PP route usually lost ≤ 22 wt % (≤9 wt % for Gd_2_O_3_:Eu). Therefore, for this route we assume that we had more complete reactions with losses mainly in the processing steps. For the UBHP route the losses vary between 25 and 69 wt %. Sample NS129 was the exception; it yielded only 5 wt % of the theoretical amount. For this sample, the relatively small amount of solid was already observed at the end of the precipitation step. As it has the smallest concentration ratio, *c*_urea_/*c*_metals_, an incomplete reaction is expected. When a 2:8 mixture of ethylene glycol is used as the synthesis medium at a low *c*_urea_/*c*_metals_ ratio of 6.3, no solid is precipitated. Thus, for the other UBHP samples an incomplete reaction conversion likely contributes, along with processing losses, to the larger differences between the theoretical and practical yield.

For the PP precipitated from this work, the composition RE_2_(O_2_)*_x_*_−*y*_(CO_3_)*_y_*(OH)_6−2*x*_·*z*H_2_O is determined from the following observations:

- There were peroxide groups detected in the evolving O_2_ by thermogravimetry coupled with mass spectrometry (TG/MS) and by the test indicating the presence of peroxide anions by the yellow-brown colour of [TiO_2_ aq.]^2+^ upon addition of aqueous TiOSO_4_-solution. A peroxidic composition can also be expected from the reaction deployed.
- The carbonate fraction is concluded from emerging CO_2_ in TG/MS and from carbon found by C/H/N/S combustion analysis. Carbonate arises by the reaction of the peroxidic fraction with CO_2_ from the air during storage.
- Hydroxide can also be expected based on the precipitation reaction and provides charge neutrality. A precipitate composition without hydroxide (but with more associated water) can be excluded by comparison of the theoretical and measured weight losses and the ratio of released H_2_O and CO_2_ upon calcination.
- Associated/adsorbed water is usually present in and on precipitates with high surface areas, here as a result of the reaction conditions and by the adsorption of water from the air.

The composition RE_2_(O_2_)_0.5_(CO_3_)_0.5_(OH)_4_·H_2_O (i.e., *x* = 1, *y* = 0.5, *z* = 1) is a compromise fitting to preliminary quantitative data concerning the weight loss in TG and concerning the H_2_O and CO_2_ contents. From this composition, the theoretical weight loss upon oxide formation adds up to 26.6 wt % for (RE = Y_0.95_Eu_0.05_), confirmed by two TG measurements up to 1000 °C (25.8 and 27.3 wt %). The precipitate with the given composition has a large hydroxide fraction and a low peroxide fraction. Collecting quantitative data from all methods was not pursued as it is difficult for the following reasons: The degree of carbonation, *y*, depends on the storage time and possibly depends on the preliminary carbonation of the Na_2_O_2_ used in the reaction. The rate of carbonation most likely increases after complete drying. It is almost impossible to control the amount of absorbed water on the nanoparticles while transferring them to the various test equipment. The specific surface area of the precipitate with RE = Y_0.95_Eu_0.05_ amounts to 286 m^2^/g (224 m^2^/g for RE = Y_0.475_Gd_0.475_Eu_0.05_ and 209 m^2^/g for RE = Gd_0.95_Eu_0.05_). These numbers correspond to precipitate particles with dimensions of a few nanometres. Thus, in the PP reaction many seeds are formed and the degree of particle growth is rather low.

In contrast, in the UBHP reaction there is slow growth of a few seeds, therefore sub-µm sized spheres are obtained with correspondingly small specific surface areas, e.g., of 4.1 m^2^/g for the case of batch NS133, the largest UBHP particles obtained in this study. In literature the product of the UBHP reaction is given as RE(OH)(CO_3_)·*z*H_2_O with 1 < *z* < 1.4 [10,17]. Theoretical weight losses for RE = Gd_0.94_Eu_0.06_ are 28.2 wt % for *z* = 1 and 30.2 wt % for *z* = 1.4. Upon calcination to 850 °C we observed values between 28.6 and 32.5 wt %. Some of the weight losses that are slightly higher than for *z* = 1.4 can be explained with adsorbed water, as no extra drying was performed in the storage period between freeze drying and calcination.

### 3.2. Size, Morphology, and Structure of the Calcined Particles

**Figure 2:**
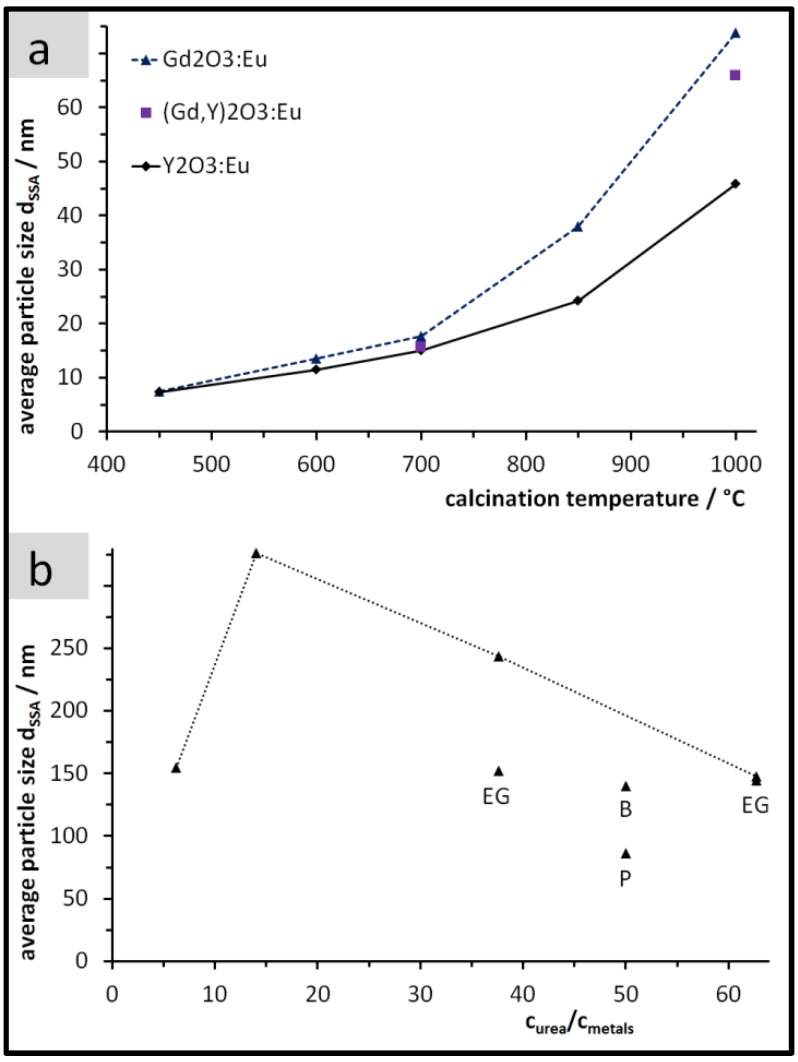
Average particle size *d*_SSA_ after calcination. (**a**) Peroxidic Precipitation (PP) particles as a function of the calcination temperature; (**b**) Urea based homogeneous precipitation (UBHP) particles after calcination at 850 °C as a function of the concentration ratio *c*_urea_/*c*_metals_ and of the reaction medium (pure H_2_O for points connected with the dotted line; others: H_2_O plus EG = ethylene glycol, B = 1-butanol, P = 2-propanol, see Table 1. At *c*_urea_/*c*_metals_ = 63 there are two overlapping data points).

After calcinating the PP solids at 450 °C they exhibited a specific surface area of 164 m^2^/g for Y_2_O_3_:Eu and of 109 m^2^/g for Gd_2_O_3_:Eu. Using Equation (1) and the densities (5.0 and 7.4 g/cm^3^ for Y_2_O_3_:Eu and Gd_2_O_3_:Eu, respectively), we obtained the result of *d*_SSA_ = 7.3 and 7.4 nm for Y_2_O_3_:Eu and for Gd_2_O_3_:Eu, respectively. Applying higher calcination temperatures generally leads to larger particles with more growth for Gd_2_O_3_:Eu in comparison to Y_2_O_3_:Eu (Figure 2a).

In contrast, the calcination of UBHP particles is only used to transform the precipitated solid into the oxide phase, it is not used to control the particle size, as the original particles are too large to do so at reasonable temperatures. Actually, the sub-µm precursor spheres shrink upon calcination as they release CO_2_ and H_2_O from their carbonate and hydroxide groups [22]. Slight reductions of the surface area are observed due to the calcination of the UBHP particles. For example, during calcination at 850 °C, batch NS133 reduces its specific surface area from 4.1 to 2.5 m^2^/g. This is because the particles undergo a transformation from amorphous material to crystalline and in doing so the surface becomes more organized and the small pores are closed.

Figure 2b depicts the size variations of UBHP particles after calcination which were realised in the precipitation stage by the concentration ratio *c*_urea_/*c*_metals_ and the variation of the solvents. For five of the tried synthesis conditions, the size of the calcined UBHP particles ends up in the range *d*_SSA_ = 140 to 155 nm. By precipitating in pure water at intermediate values of the concentration ratio, larger particles (*d*_SSA_ = 326 and 244 nm) are obtained. To synthesise the largest particles of this work, we followed the results of Zhu et al. [9] that describes a maximum particle size at smaller *c*_urea_/*c*_metals_ values in the explored range. The smallest particles of our work (*d*_SSA_ = 86 nm) were achieved with a fraction of 2-propanol in the synthesis medium [22].

Altogether, for Gd_2_O_3_:Eu particles and based on the size information *d*_SSA_, for PP particles we achieved a variation between 7 and 74 nm (7–46 nm for Y_2_O_3_:Eu) and between 86 and 326 nm for UBHP particles. For the commercial Y_2_O_3_:Eu particles ‘L581’, we obtained the size information *d*_SSA_ = 2.3 µm based on our own SSA measurement.

**Figure 3:**
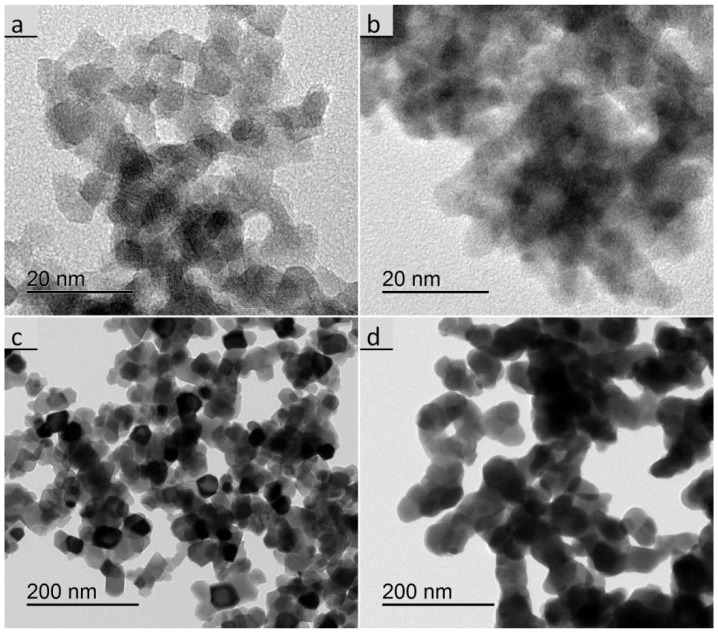
Transmission electron microscope (TEM) images of the PP particles, (**a**) Y_2_O_3_:Eu calcined at 450 °C; (**b**) Gd_2_O_3_:Eu calcined at 450 °C; (**c**) Y_2_O_3_:Eu calcined at 1000 °C; (**d**) Gd_2_O_3_:Eu calcined at 1000 °C.

XRD analyses were made with PP particles at the calcination states of 450 °C and 1000 °C, as well as with most of the calcined UBHP particles. The cubic RE-oxide phase could be assigned in all cases and there were no peaks in the diffractograms pointing to other phases. The diffractogram of the Gd_2_O_3_:Eu sample calcined at 450 °C differs from all others, as it has relatively low intensities and with only the four main peaks of the cubic phase arising slightly with a broad shape from the elevated background. Thus, it can be concluded that there was incomplete crystallisation resulting in very small crystallites (*d*_XRD_ = 2.3 nm), which is in agreement with the morphology visualized in the TEM (Figure 3b). In contrast, the Y_2_O_3_:Eu sample calcined at 450 °C has distinct particles that are easily visible in the TEM (Figure 3a) micrographs and the sizes from different techniques are in agreement (*d*_SSA_ = 7.3 nm, *d*_TEM_ = 6 nm, *d*_XRD_ = 7.4 nm).

For the 1000 °C calcination the associated sizes *d*_SSA_ = 46 nm, *d*_TEM_ = 37 nm, *d*_XRD_ = 43 nm are sufficiently in agreement for Y_2_O_3_:Eu. The Gd_2_O_3_:Eu particles appear larger and more aggregated in the TEM images than the Y_2_O_3_:Eu particles (Figure 3c,d). For the Gd_2_O_3_:Eu particles we get *d*_SSA_ = 74 nm, which is significantly higher than *d*_TEM_ = 45 nm and *d*_XRD_ = 43 nm. This difference confirms that there is a larger degree of aggregation as the connection of the particles via thick sintering necks reduces the surface area resulting in a larger *d*_SSA_ size. Generally, the crystallised particles are equiaxed, for Y_2_O_3_:Eu they mostly exhibit flat crystal surfaces and Gd_2_O_3_:Eu particles appear more round shaped.

**Figure 4:**
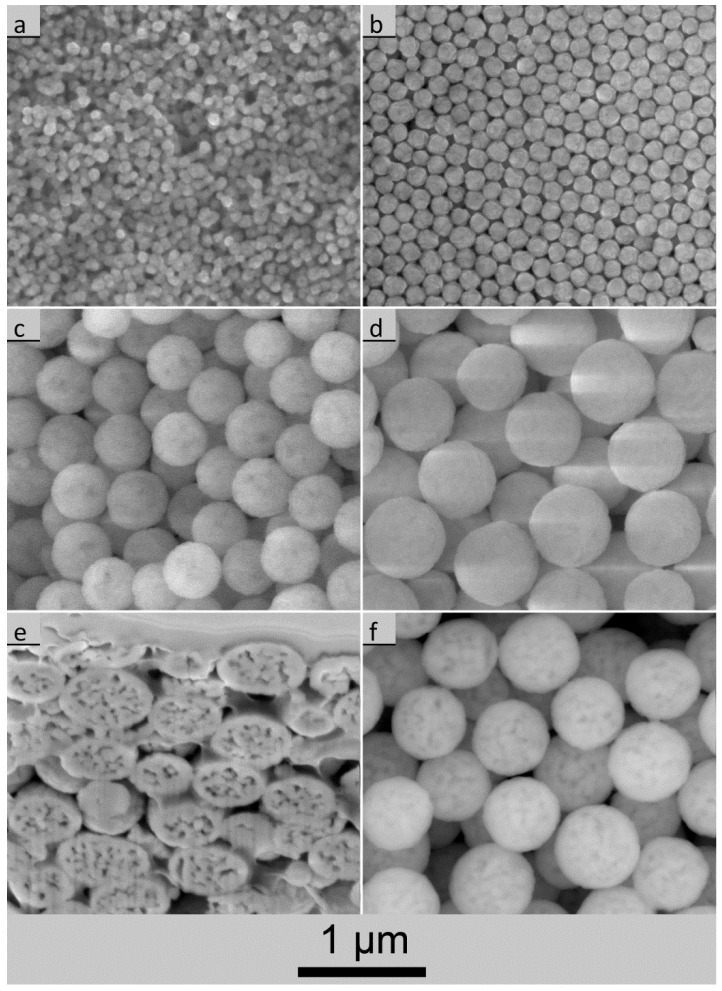
Environmental scanning electron microscope (ESEM) images [taken with the large field detector, exception: image (f)] of UBHP Gd_2_O_3_:Eu particles calcined at 850 °C; (**a**) NS141-850 (*d*_SSA_ = 86 nm); (**b**) NS130-850 (*d*_SSA_ = 147 nm); (**c**) NS126-850 (*d*_SSA_ = 244 nm); (**d**) NS133-850 (*d*_SSA_ = 326 nm); (**e**) NS133-850—view into a trench cut by Focused Ion Beam (FIB) into the pellet pressed for prior cathodoluminescence (CL) examination; (**f**) NS133-850—particle image, taken with the back-scattered electron (BSE) detector.

In Figure 4a–d the variety of UBHP particle sizes and morphologies is shown by ESEM images which were taken with the large field detector. The spherical shape with a narrow size distribution is typical for UBHP particles. During calcination the UBHP particles have a low number of neighbour contacts due to prior freeze drying (very loose particle packing) and due to the shrinkage taking place during heating. At the contacts, sintering necks are forming which have a small diameter in comparison to the large particles. Only for the small particles depicted in Figure 4a does more aggregation become visible. Due to the polycrystalline structure (see below), facets and edges emerge for the smaller particles, and are especially well represented in Figure 4b. In contrast, the larger particles appear as spheres with rough surfaces. Depending on contrast settings and charging, in some of the LFD images the particles exhibit dark areas (Figure 4c), but in other LFD images this effect cannot be seen (Figure 4d). The SEM image of a focused ion beam (FIB) cut through the particles reveals that these dark areas are caused by pores lying under dense surface shells (Figure 4e). The polycrystalline and porous structures were described by Vecht et al. as tessellated crystallites [32]. In a few particles, open connections from the inside porosity to the surface were revealed by the FIB cut. Furthermore, imaging with the backscattered electron (BSE) detector refines the contrast from the underlying pore structure (Figure 4f) in comparison to the low contrast LFD images (Figure 4d). The described pore structure can be traced back to the decarbonation and the dehydroxylation of the UBHP precipitates during calcination. After the formation of the porous core–shell structure, the calcination conditions (6 h at 850 °C) are obviously not sufficient to densify the particles completely. The unique porosity of UBHP particles is also described by Bezkrovnyi et al. [13].

**Figure 5:**
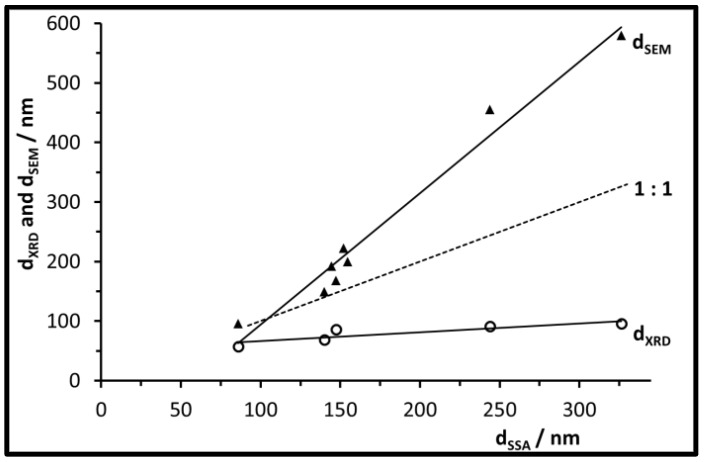
Particle size *d*_SEM_ and crystallite size *d*_XRD_ of the calcined UBHP particles, as a function of the size information *d*_SSA_.

The plot in Figure 5 compares the different sizes reported by the different characterization techniques for the UBHP particles. Their crystallite sizes increase slightly with increasing particle sizes. Thus, small particles consist of few crystallites (*d*_SSA_ = 86 nm, *d*_XRD_ = 57 nm, *d*_SEM_ = 96 nm) whilst the large ones consist of a large number of crystallites (*d*_SSA_ = 326 nm, *d*_XRD_ = 96 nm, *d*_SEM_ = 580 nm). All particle sizes analysed by SEM images are larger than those calculated from the specific surface area. For the particles with sizes up to *d*_SSA_ = 155 nm this can be explained with the surface roughness which leads to larger surface areas in comparison to smooth spheres. This also applies for the two batches of larger spheres, but their size ratio *d*_SEM_/*d*_SSA_ becomes larger based on extra surface area contributed by the pore walls in the inside of the particles. This inner porosity only provides extra area for N_2_ adsorption (BET method) if it is connected by open pore channels to the particle surface. Based on the FIB cut described above, the ratio *d*_SEM_/*d*_SSA_ for large particles, and the CO_2_ + H_2_O degassing during calcination, such an accessible porosity can be determined. Thus, to describe the larger particles correctly with respect to geometric dimensions, the *d*_SEM_ information is more appropriate. However, since surface area effects can support mechanisms known to quench chromophores, we will keep on using *d*_SSA_ as the main diameter descriptor in the following sections.

### 3.3. Luminescence of the Particles: Spectra, QY, and Relative Emission Light Intensities

**Figure 6:**
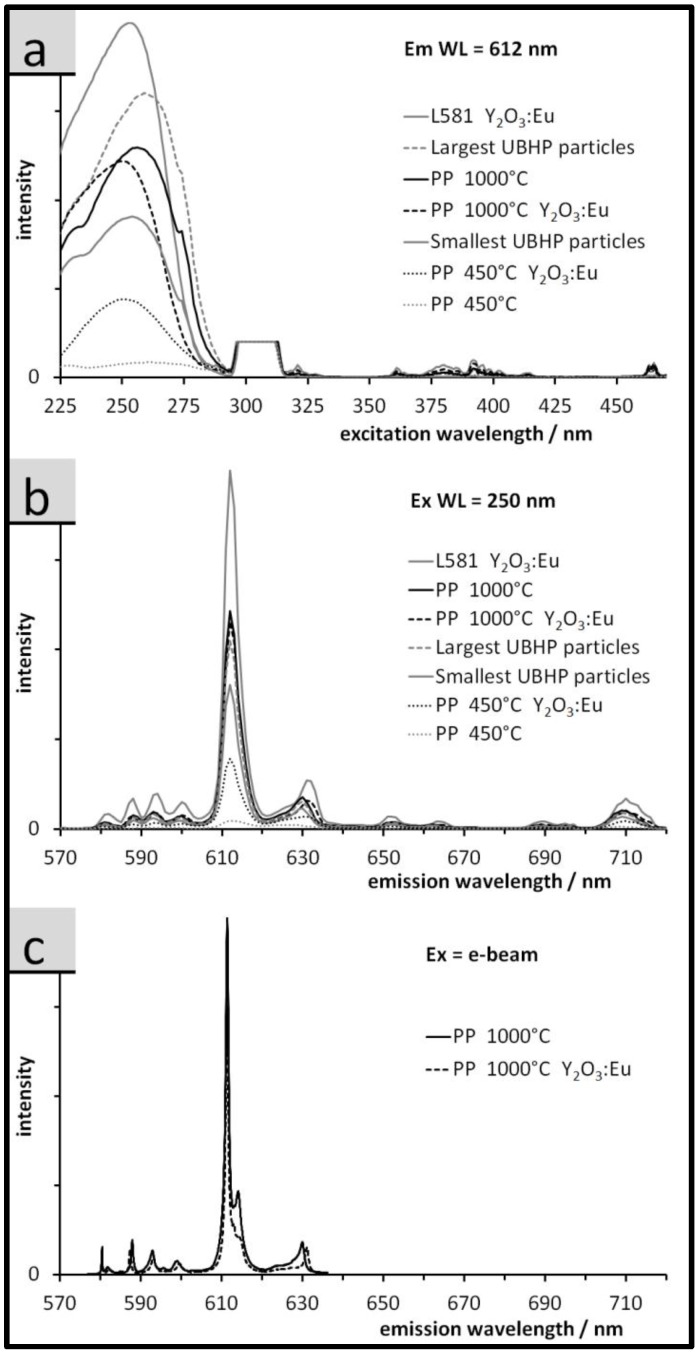
Luminescence spectra of the Gd_2_O_3_:Eu particles (no indication) and of the Y_2_O_3_:Eu particles (where indicated), (**a**) fluorescence excitation spectra (cut peak around 306 nm = 1st order diffraction from monochromator, i.e., spectrometer artefact); (**b**) fluorescence emission spectra; (**c**) cathodoluminescence emission spectra (taken from 577 to 636 nm).

The photoluminescence excitation (PLE), photoluminescence (PL), and cathode luminescence (CL) spectra are displayed in Figure 6a–c respectively. The visible fluorescence in Eu^3+^ doped RE oxides arises due to the unique electronic structure of the Eu^3+^ dopant. The Eu 4*f* orbitals are partially filled and are substantially screened by the 5*s* and 5*p* orbitals from the local field of the host matrix. This screening diminishes the effect of the crystal field on the 4*f* states allowing Judd-Ofelt (J-O) radiative transitions within the free ion like dopant, resulting in spectrally narrow absorption and emission bands. However, the Eu^3+^ ion has a very small absorption cross section, therefore, direct excitation is a low probability event. The more probable excitation processes involve down conversion of photo or cathode excited electrons from the large band gap RE oxide. Specifically, the photo or cathode excited electrons in the RE oxide non-radiatively relax to an energy level that allows a charge transfer (CT) process to the Eu^3+^ 4*f* J-O active levels. The possible excitation transitions include excitonic transitions (ET) of the host (O_2p_ → Y, Ga), populating the CT levels of the Eu^3+^ (O_2p_ → Eu), or the higher lying *f*–*f* levels of the Eu^3+^. The optically active J-O transitions can be assigned as electric dipole and magnetic dipole allowed Eu^3+^ ^5^D_0_ → ^7^F*_J_* (*J =* 1–4) transitions [33].

The PLE spectra are displayed in Figure 6a and are monitored at the ^5^D_0_ → ^7^F_2_ emission at 612 nm (Em wavelength). The spectra are dominated at short wavelengths by two high energy peaks, the excitonic peak at 210 nm for Y_2_O_3_ and 230 nm for Gd_2_O_3_, and the O_2p_ → Eu_4f_ charge transfer (CT) transition at 240 nm for Y_2_O_3_ and 250 nm for Gd_2_O_3_ [33,34]. The peak at 306 nm is a result of a 1^st^ order diffraction artefact of the monochromator. The lower energy transitions between 350 and 475 nm are assigned to the Eu^3+^ transitions from ^7^F_0_ → D*_i_* (*i =* 1–3).

The spectral line shapes are similar for the samples not represented in Figure 6. In Figure 6, the spectra differ strongly in intensities; L581 Y_2_O_3_:Eu has the highest intensity and the dominant peak at 240 nm indicates there is efficient energy transfer to the Eu^3+^. However, as the particle size decreases, the ET peaks for both hosts become resolved indicating a reduction in energy transfer to the Eu^3+^ [35]. The Gd_2_O_3_:Eu sample from the PP synthesis, calcined at 450 °C, has almost no CT band and the ET transition is very weak indicating the host matrix is poorly organized and the energy transfer is very inefficient. The XRD results confirm the formation of the cubic (luminescent) RE-oxide phases in all cases but with incomplete crystallisation, resulting in very small crystallites for the Gd_2_O_3_:Eu sample calcined at 450 °C.

The emission spectra (upon excitation at 250 nm, Figure 6b) are characteristic of the Eu^3+^ dopant. The ^5^D → ^7^F_2_ transition indicates that the Eu^3+^ is in a site in the host lattice with low symmetry without inversion; it is at 612 nm, with a secondary peak at 630 nm due to stark splitting. The peaks between 585 and 603 nm (^5^D_0_ → ^7^F_1_) indicate the S6 and C3 lattice positions are populated [36], and for all samples the Eu^3+^ dopant is only emissive from the ions located in the interior of the particle as seen by the ^5^D_0_ → ^7^F_0_ transition located at 581 nm [37]. The peaks from 640 nm to 725 nm are assigned to the ^5^D_0_ → ^7^F_2,4_ transition and taken together with the ^5^D_0_ → ^7^F_1,2_ transitions indicate that the host matrix, except for PP 450 °C Gd_2_O_3_, is well crystallized. The spectra in Figure 6c show a smaller spectral range of emissions, excited by a SEM electron beam. Here the same peaks emerge as seen in the PL spectra, however, they are narrower due to the improved spectral resolution of the grating used.

**Figure 7:**
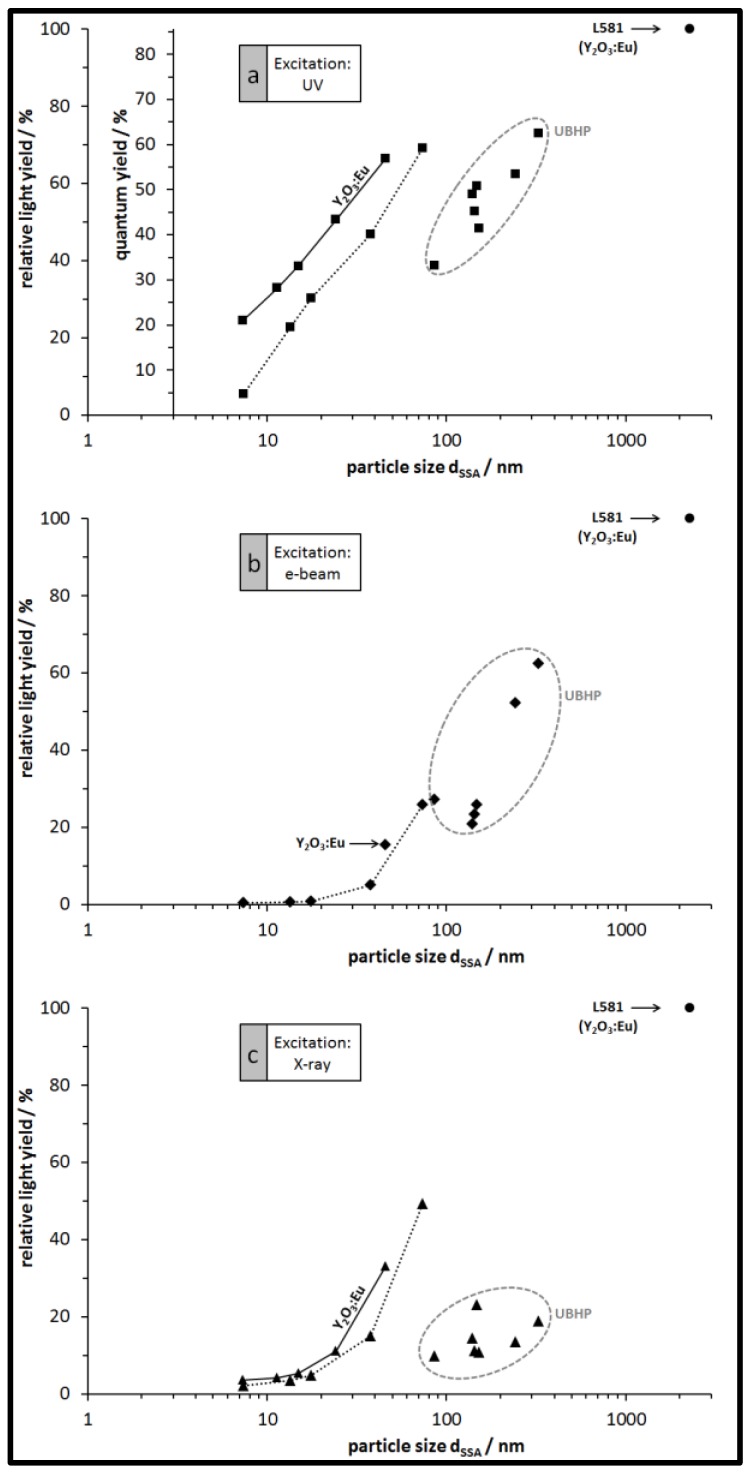
Relative light yield of the Gd_2_O_3_:Eu particles (no indication) and of the Y_2_O_3_:Eu particles (where indicated), depicted in dependence of the particle size *d*_SSA_. Particles with sizes up to 74 nm are from the PP synthesis, larger particles with data points in the ovals are from the UBHP synthesis. (**a**) For excitation with 250 nm UV light (second ordinate giving the absolute QY values); (**b**) for excitation with e-beam (cathodoluminescence); (**c**) for excitation with X-ray.

The relative light yield for each particle type plotted against the particle size and for the three excitations of the luminescence is shown in Figure 7. For each excitation type, the light yield of the largest L581 particles is set to 100% and all other particles from our syntheses emit less light. For all particles from the PP synthesis and for all excitations there is a clear trend: larger particles (resulting from higher calcination temperatures) generally emit more light than smaller particles. The emission light of Y_2_O_3_:Eu particles is a bit brighter than that of the Gd_2_O_3_:Eu particles as expected. Within the group of UBHP particles there is a bit more scatter in the data, nevertheless, they exhibit the same trend of increasing emitted light intensity with increasing particle size and decreasing SSA. However, with respect to the size of the UBHP particles, they have lower emission intensity than the extrapolated trends of the PP particles would imply. This suggests that there is a surface area related mechanism active that is quenching the emission. Making a reasonable assumption that the surfaces of the particles have many recombination sites, the observed changes in the PLE spectra with particle size becomes understandable. As noted previously, as the particle size decreases, the ET and CT bands also decrease; they are quenched by surface recombination since the incident light has a penetration depth of 100 nm [35,38]. We also noted that there is no emission from dopants at or near to the particle surface, further implying there is no CT to the dopants on or near the surface. There are also reports that the vibrations of surface bound water molecules can interact with electrons situated in the long lived ^5^D_0_ state through a competing multiphonon non-radiative emission process. The distance of this interaction is most likely similar to the energy transfer between two dopants about 6 Å [35,39].

As noted previously the UBHP particles are not as luminescent as their size would predict. This is interesting because unlike the PP particles, all of the UBHP particles have been calcined at the same temperature (850 °C). They all have the same dopant level (6 mol %) and the particle size was achieved by varying the *c*_urea_/*c*_metal_ ratio. Compared to the PP synthesized particles, the UBHP particles have additional internal surface area (Figure 4e). This internal surface area may not be accessible to water after calcination. However, since most of the batches have *d*_SSA_ ca. 150 nm they are certainly penetrated by the incident light, yet, they are not as luminescent as the PP particles. Schmechel et al. reported a similar quenching effect in Y_2_O_3_:Eu caged in aerogels [35]. They proposed that there are phonons excited in the pore walls which take up the energy of the electrons in the long lived ^5^D_0_ state. This extra source of phonons could account for the discrepancy in luminescence seen in the porous UBHP particles.

## 4. Conclusions

This is the first study covering luminescence intensity measurements on Y_2_O_3_:Eu and Gd_2_O_3_:Eu powder samples for a broad particle size range and for the application of three excitation sources (UV, e-beam, and X-ray) on the same set of particles. In this study, the relative light intensity values for the UV excitation are based on real quantum yield measurements. These are applied seldomly as it is more usual to compare count levels of spectra which do not consider different UV absorption grades by different powders.

We also present a new synthesis route for RE_2_O_3_:Eu particles based on peroxidic precipitation. After calcination of the precursor phase, the particles display the typical luminescence properties exhibited by RE oxide particles produced from other precipitation chemistries. Different calcination temperatures were used to obtain different sizes of PP particles.

To produce a range of sub-µm size particles, a UBHP reaction was employed at different precipitation conditions, followed by the same calcination temperature of 850 °C. The porous and polycrystalline character of the UBHP particles is described in detail using FIB milling and SEM imaging for the first time.

From the combined results obtained with three different excitation methods, it can be concluded that the smaller particles emit less light than the larger particles. This trend is experimentally covered with the PP particles down to a particle size of 7 nm. Therefore, even the smallest of our particles do not give evidence that quantum confinement could improve luminescence performance. In contrast, studies have shown that the large surface area of nanoparticles host recombination centers and have adsorbates like water which quench luminescence [4]. The proposed effect of multiphonon emission competing with down conversion processes due to increased surface areas external and internal to the particles explains the decrease in luminescence as the particle size decreases.

The set of UBHP particle types which were all calcined at the same temperature gives a confirmation for a real size effect and excludes a potential hypothesis that the emission intensity predominantly depends on the calcination temperature, e.g., by decreasing defect levels in the crystal lattices with increasing calcination temperature. Therefore, a predominant size effect can be concluded for the particles from both synthesis routes.

## Figures and Tables

**Table 1:** Varied parameters of urea based homogeneous precipitation (UBHP) syntheses.

Batch	NS129	NS133	NS126	NS130	NS141	NS134	NS127	NS132
Solvent [vol.-ratio]	H_2_O	H_2_O	H_2_O	H_2_O	2-propanol + H_2_O (6:4)	1-butanol + H_2_O (6:4)	ethylene glycol + H_2_O (2:8)	ethylene glycol + H_2_O (2:8)
*c*_urea_/*c*_metals_	6.3	14.1	37.6	62.7	50.1	50.1	37.6	62.7
